# Development and comparison of machine learning-based models for predicting heart failure after acute myocardial infarction

**DOI:** 10.1186/s12911-023-02240-1

**Published:** 2023-08-24

**Authors:** Xuewen Li, Chengming Shang, Changyan Xu, Yiting Wang, Jiancheng Xu, Qi Zhou

**Affiliations:** 1 Department of Laboratory Medicine, First Hospital of Jilin University, Changchun, China; 2 Information center, First Hospital of Jilin University, Changchun, China; 3 Medical Department, First Hospital of Jilin University, Changchun, China; 4 Department of Pediatrics, First Hospital of Jilin University, 1Xinmin Street, Changchun, 130021 Jilin China

**Keywords:** Acute myocardial infarction, Heart failure, Machine learning, Extreme gradient boosting, Model

## Abstract

**Aims:**

Heart failure (HF) is one of the common adverse cardiovascular events after acute myocardial infarction (AMI), but the predictive efficacy of numerous machine learning (ML) built models is unclear. This study aimed to build an optimal model to predict the occurrence of HF in AMI patients by comparing seven ML algorithms.

**Methods:**

Cohort 1 included AMI patients from 2018 to 2019 divided into HF and control groups. All first routine test data of the study subjects were collected as the features to be selected for the model, and seven ML algorithms with screenable features were evaluated. Cohort 2 contains AMI patients from 2020 to 2021 to establish an early warning model with external validation. ROC curve and DCA curve to analyze the diagnostic efficacy and clinical benefit of the model respectively.

**Results:**

The best performer among the seven ML algorithms was XgBoost, and the features of XgBoost algorithm for troponin I, triglycerides, urine red blood cell count, γ-glutamyl transpeptidase, glucose, urine specific gravity, prothrombin time, prealbumin, and urea were ranked high in importance. The AUC of the HF-Lab9 prediction model built by the XgBoost algorithm was 0.966 and had good clinical benefits.

**Conclusions:**

This study screened the optimal ML algorithm as XgBoost and developed the model HF-Lab9 will improve the accuracy of clinicians in assessing the occurrence of HF after AMI and provide a reference for the selection of subsequent model-building algorithms.

## Introduction

Acute myocardial infarction (AMI) is associated with nursing acute syndrome caused by a sharp blockage of the coronary arteries. AMI causes massive necrosis of cardiomyocytes, which are non-renewable cells, and the more cardiomyocytes that die, the more severe the damage to the heart. Destruction of myocardial cells and scar proliferation activate the neurohumoral system and cause ventricular remodeling [1]. Cardiac remodeling will lead to enlargement of the heart and a significant decrease in contractility. When the heart contracts, the amount of blood expelled will be significantly reduced and a large amount of blood will accumulate in the heart. When the heart cannot bear it, the blood flows upward back to the lungs, causing the patient to suffer from chest tightness and difficulty breathing, which is a serious threat to life. Approximately 13% of AMI patients are diagnosed with HF 30 days after discharge, and 20–30% of AMI patients are diagnosed with heart failure (HF) 1 year after discharge [2, 3]. HF occurs in about a quarter of AMI patients and is a major cause of increased mortality [4]. The European Society of Cardiology [5] and the American Heart Association [6] indicate that the prevention of HF is an urgent public health need. The post-AMI patient population is at high risk for the development of HF, of which HF screening and prevention are particularly important. Missed or delayed diagnosis of HF can jeopardize a patient prognosis and increase the cost of treatment. Therefore, it is essential to explore effective methods and markers for the development of HF after AMI, and early identification of patients with a high risk of poor prognosis can save lives and improve the quality of patient survival through personalized treatment [7].

Traditional ML algorithms such as Logistic Regression (LR), Decision Tree (DT), and Random Forest (RF) have been used with good success in various fields of medicine. For example, the use of LR to identify heart failure in patients with coronary heart disease [8]; DT analysis to identify patients at risk of death or hospitalization due to worsening heart failure [9]; RF classifier to detect congestive heart failure [10]. In recent years, ML algorithms other than traditional ML algorithms have increasingly penetrated into various medical fields, such as extreme gradient boosting (Xgboost) methods have shown superior diagnostic capabilities among many ML algorithms [11]. Then the advantages of various ML algorithms may differ in different diseases and different statistical contexts. Before building a model, the best and most appropriate algorithms should be evaluated and compared so that clinicians can provide better healthcare to patients. A large amount of patient test information resides in the healthcare system, and the value of this test information should not stop at surface abnormal values. ML can explore the potential connections in a large amount of test information and uncover the “deep language” to provide higher value for disease prediction and diagnosis. In this study, we propose to use seven ML algorithms to develop an early warning model that can accurately predict the risk of HF after AMI from a large amount of test information, and compare the prediction performance of the seven algorithms in order to provide a reliable method and biomarker for predicting HF after AMI, which can help improve the prognosis and survival quality of patients.

## Materials and methods

### Study design and subject statistics

Study participants were recruited from the First Hospital of Jilin University and divided into 2 cohorts: (1) Algorithm and feature selection of HF early warning model (cohort 1): patients with confirmed AMI from January 2018 to December 2019, divided into HF group and non-HF group (control group); (2) HF early warning model development and comparison (cohort 2): patients with confirmed AMI from January 2020 to December 2021, were also divided into HF and control groups. Inclusion criteria for patients with AMI: (a) age > 18 years; (b) first diagnosis of AMI at admission, including clinical symptoms, typical changes in the electrocardiogram, are elevated cardiac biomarkers, in accordance with the current guidelines for the diagnosis of AMI [12]; (c) No HF on admission. Exclusion criteria: (a) pulmonary fibrosis or other serious diseases that prevented image acquisition (massive pleural effusion, severe emphysema, lung cancer, etc.); (b) non-obstructive myocardial infarction; (c) serious immune system diseases; (d) malignant tumors with malignant hematological diseases; (e) serious infections; (f) death during hospitalization. The HF group included patients with AMI who developed HF during hospitalization and patients with AMI who were readmitted for HF after discharge from the hospital. This study was approved by the institution’s ethics committee of the First Hospital of Jilin University (2016–306).

### Data cleansing and normalization

Demographic and case information (age, gender, medical history) for all participants were obtained from the medical record system. Extracting the first test data (routine blood test, routine urine test, coagulation function, liver function, kidney function, lipids, blood glucose, cardiac protein, BNP, and other tests) from the laboratory information system after the study subjects were admitted to the hospital, and each study subject corresponds to a unique ID number. (a) data cleaning: exclude patients with missing test data or outliers. A normality test is performed on continuous variables, a padding method (median, mean, or plurality) that reflects the central characteristics of the variable is chosen for missing value padding, and the differential analysis of the performance of models built before and after data interpolation to assess the robustness of the interpolation method; (b) data normalization: data are normalized according to four parameters: the origin of the specimen, name of the test item, the unit of the test item, and reference interval.

### ML algorithms and model building

The cohort 1 data set is divided into 5 folds by 5-fold cross-validation, and 4 of the folds are used as the training set to train the model, and the remaining 1 fold is used as the validation model, which is repeated 5 times to take the average value. The algorithms used are Xgboost, random gradient descent (RGD), linear support vector classification (linear SVC), Adaptive Boosting (AdaBoost), LR, RF, and DT, which are seven common and filterable features. ML algorithms to build early warning models of HF and compare the results of internal 5-fold cross-validation of the 7 classifiers. Based on the comparison results, the optimal ML algorithm is selected as the subsequent algorithm for model building and validation, and the selected features of the model are screened.

The cohort 2 dataset is divided into training and validation sets in the ratio of 7:3, and the post-AMI HF prediction model is built based on the optimal algorithm and model features screened in Cohort 1 and externally validated.

### Statistical analysis

Excel 2016, SPSS 22.0, and GraphPad 8.0.2 were utilized for data management and statistical analysis. Feature selection and model development were performed using the Deepwise & Beckman Coulter DxAI platform (http://dxonline.deepwisecom). Receiver operating characteristic (ROC) curves assessed the predictive capability of the HF model, while decision curve analysis (DCA) evaluated its clinical benefits. Continuous variables were subjected to normality tests; normally distributed variables were presented as mean ± standard deviation, and non-normally distributed variables as median (*Q*_25_, *Q*_75_). Comparisons of variable distributions between groups were conducted using Student’s t-test, Mann-Whitney U test, ANOVA, or Kruskal-Wallis H test. Categorical variables were expressed as composition ratios, with Pearson’s χ² test or Fisher’s exact test employed for comparing distributions between groups. A two-sided P-value of < 0.05 was considered to indicate a statistically significant difference.

## Results

### Enrolled population and demographic characteristics

A total of 3312 patients with AMI were included in this study, cohort 1 contained 1625 cases, 801 (49.3%) in the HF group and 824 (50.7%) in the control group; cohort 2 contained 1687 cases, 820 (48.6%) in the HF group and 867 (51.4%) in the control group. There was no statistically significant difference in the distribution of study subjects between cohort 1 and cohort 2 (*P* = 0.780). Both cohort 1 and cohort 2 exhibited significant differences in age (P < 0.001) and gender (P < 0.001) between the HF and control groups. Consequently, age and gender were included as characteristics for selection in the HF early warning model. The hypertensive population percentage in the HF group was 52.3% and 52.8% in cohorts 1 and 2, respectively, with no statistically significant difference compared to the control group (Table 1). The diabetic population percentage was 17.6% and 19.1%, respectively, and the difference compared to the diabetic population percentage in the control group was not statistically significant (Table 1).

**Table 1:** Demographic characteristics of the subjects

Items	All enrollees (n = 3312)	Cohort 1 (n = 1625)	*P* values	Cohort 2 (n = 1687)	*P* values
All patients (%)					
HF	1621(48.9)	801 (49.3)		820(48.6)	
Control	1691(51.1)	824(50.7)		867(51.4)	0.780
Age ± SD					
HF	63.0 ± 12.0	64.0 ± 12.0		63.0 ± 12.0	
Control	61.0 ± 12.0	60.0 ± 12.0	< 0.001	61.0 ± 11.0	< 0.001
Gender ( male %)					
HF	963(59.4)	469 (58.6)		494 (60.2)	
Control	1220(72.1)	601 (72.9)	< 0.001	619 (71.4)	< 0.001
High blood pressure (%)					
HF	852(52.6)	419(52.3)		433(52.8)	
Control	849(50.2)	420(51.0)	0.620	429(49.5)	0.173
Diabetes (%)					
HF	298(18.4)	141(17.6)		157(19.1)	
Control	269(15.9)	129(15.6)	0.317	140(16.1)	0.110

### ML algorithm and model feature selection

Cohort 1 was chosen based on specific criteria: the initial test data from AMI patients after admission and test items with a missing rate of less than 30%. Out of 664 routine laboratory tests, 70 items were extracted as potential features for model selection. Median replacement interpolation, representing the central tendency of variables, was selected as the method for filling in missing values in quantitative data. Age and gender were also included as potential features due to significant differences between the HF and control groups, resulting in a total of 72 possible features for the ML model.

The internal 5-fold cross-validation results of seven algorithms revealed that XgBoost had the highest AUC (0.973), sensitivity (0.896), and specificity (0.955) in predicting HF after AMI (Table 2). Consequently, XgBoost was chosen as the algorithm for further modeling, and the top nine features with the highest feature importance in the XgBoost algorithm were utilized (For a decision tree, the feature importance is measured by the amount by which each attribute partition point improves the performance metric, weighted by the number of observations for which that node is responsible. The feature importance is averaged over all decision trees within the model.). These features included Troponin I (cTnI), Triglycerides (TG), Urine red blood cell count (URBC), Glucose (GLU), Urine specific gravity (SG), Thrombin time (TT), γ-Glutamyl transferase (γ-GT), Prealbumin (PAB), and Urea. Table 3 displays the distribution of these nine characteristics between the groups in cohort 1. The HF group exhibited significantly higher levels of cTnI, TG, GLU, γ-GT, and Urea compared to the control group (P < 0.001), while SG, TT, and PAB were significantly lower (P < 0.001).

**Table 2:** Diagnostic efficacy of Seven classifiers

Classifier	AUC [95%CI]	Sensitivity	Specifity	Accuracy	Precision	Recall	F1 score	Positive predictive value	Negative predictive value
Decision Tree	0.939 [0.9274–0.9505]	0.875	0.881	0.878	0.877	0.875	0.876	0.877	0.879
AdaBoost	0.940 [0.9289–0.9503]	0.829	0.885	0.857	0.875	0.829	0.851	0.875	0.842
Linear SVC	0.915 [0.9382–0.9584]	0.814	0.869	0.842	0.858	0.814	0.835	0.858	0.828
XgBoost	0.973 [0.9658–0.9797]	0.896	0.955	0.926	0.951	0.896	0.923	0.951	0.905
Random Forest	0.955 [0.9456–0.9646]	0.820	0.952	0.887	0.943	0.820	0.877	0.943	0.845
Random gradient descent	0.906 [0.8916–0.9204]	0.812	0.850	0.831	0.840	0.812	0.825	0.840	0.823
Logistic Regression	0.914 [0.9007–0.9281]	0.810	0.865	0.838	0.854	0.810	0.832	0.854	0.824

*Sensitiity = True Positive /( True Positive + False Negative); Specificity = True Negative/( True Negative + False Positive); Accuracy = (True Positive + True Negative)/( Positive + Negative); Precision = True Positive/( True Positive + False Positive); Recall = True Positive /( True Positive + False Negative); F1 score = = 2*Precision*Recal /(Precision + Recal); Positive predictive value = True Positive/( True Positive + False Positive); Negative predictive value = True Negative/( True Negative + False Negative)

**Table 3:** Distribution of the 9 features between groups in cohort 1 and cohort 2

Indicators	Queue 1 (n = 1625)			Queue 2 (n = 1687)		
	HF (n = 801)	Control (n = 824)	*P* values	HF (n = 820)	Control (n = 867)	*P* values
Troponin I ng/mL	20.000(11.200–29.300)	13.600(4.250-35.000)	< 0.001	20.000(11.832–29.225)	16.100(4.780-38.175)	< 0.001
Triglycerides (mmol/L)	1.800(1.520–2.190)	1.570(1.110–2.060)	< 0.001	1.825(1.540–2.272)	1.600(1.150–2.090)	< 0.001
Urine red blood cell count (10^6^/L)	6.100(2.700–6.600)	5.600(3.000-8.100)	0.866	6.200(3.200–6.900)	5.600(3.100–8.100)	0.287
Glucose (mmol/L)	7.770(7.250–8.725)	7.250(6.060–8.500)	< 0.001	8.512 ± 2.578	7.621 ± 2.123	< 0.001
Urine specific gravity	1.015(1.011–1.020)	1.027(1.017–1.046)	< 0.001	1.016(1.013–1.022)	1.028(1.018–1.045)	< 0.001
Thrombin time (s)	16.600(14.700–17.400)	17.400(16.700–18.000)	< 0.001	16.600(14.900–17.400)	17.400(16.700–18.100)	< 0.001
γ- Glutamyl transferase (U/L)	35.000(23.900–47.800)	27.150(18.875-39.000)	< 0.001	35.250(24.475–47.175)	28.100(18.850–38.950)	< 0.001
Prealbumin (g/L)	0.200(0.160–0.240)	0.240(0.210–0.270)	< 0.001	0.210(0.170–0.250)	0.240(0.210–0.270)	< 0.001
Urea (mmol/L)	6.210(5.840–8.338)	5.580(4.570–6.725)	< 0.001	6.210(6.128–8.338)	5.593(4.550–6.680)	< 0.001

*Normally distributed variables are expressed as mean ± standard deviation, and non-normally distributed variables are expressed as median (*Q*_25_, *Q*_75_)

### HF early warning model development and external validation

**Fig. 1:**
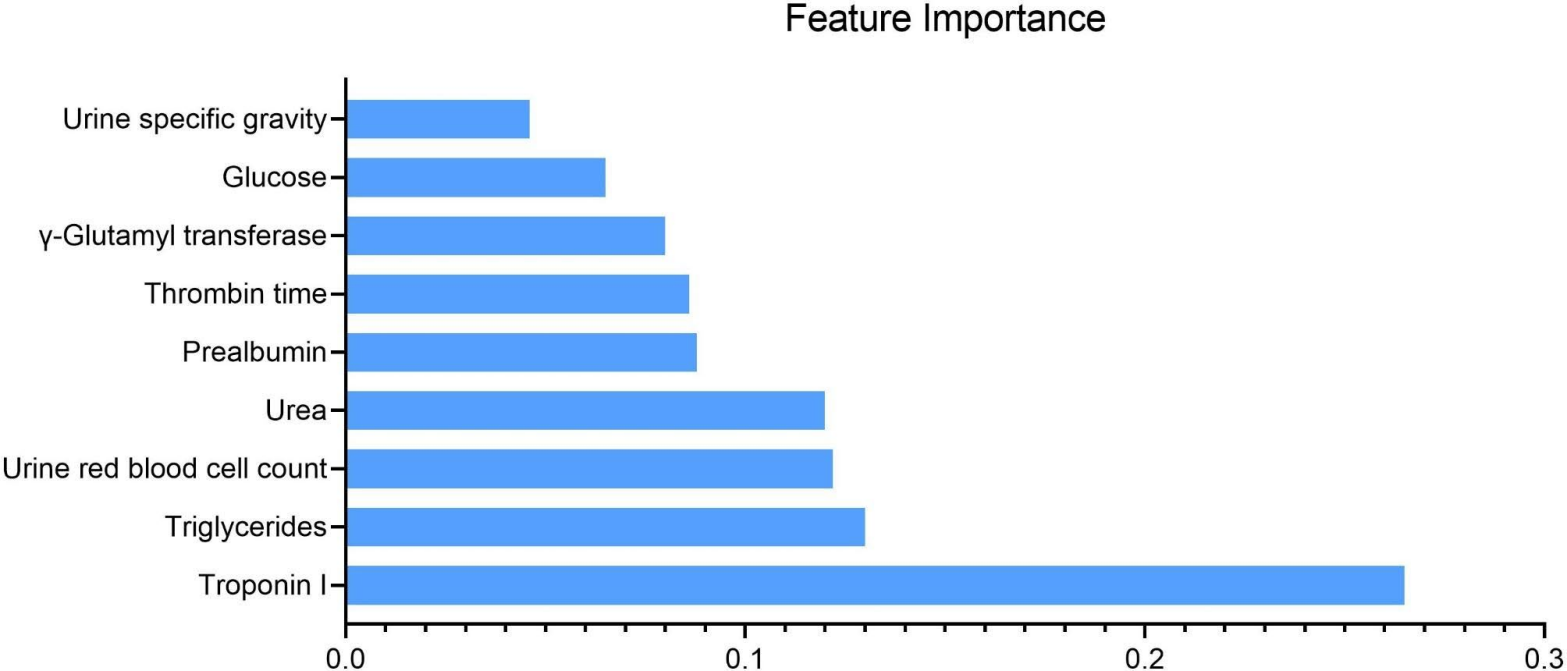
Feature importance ranking of the 9 features in the HF-Lab9 model

**Fig. 2:**
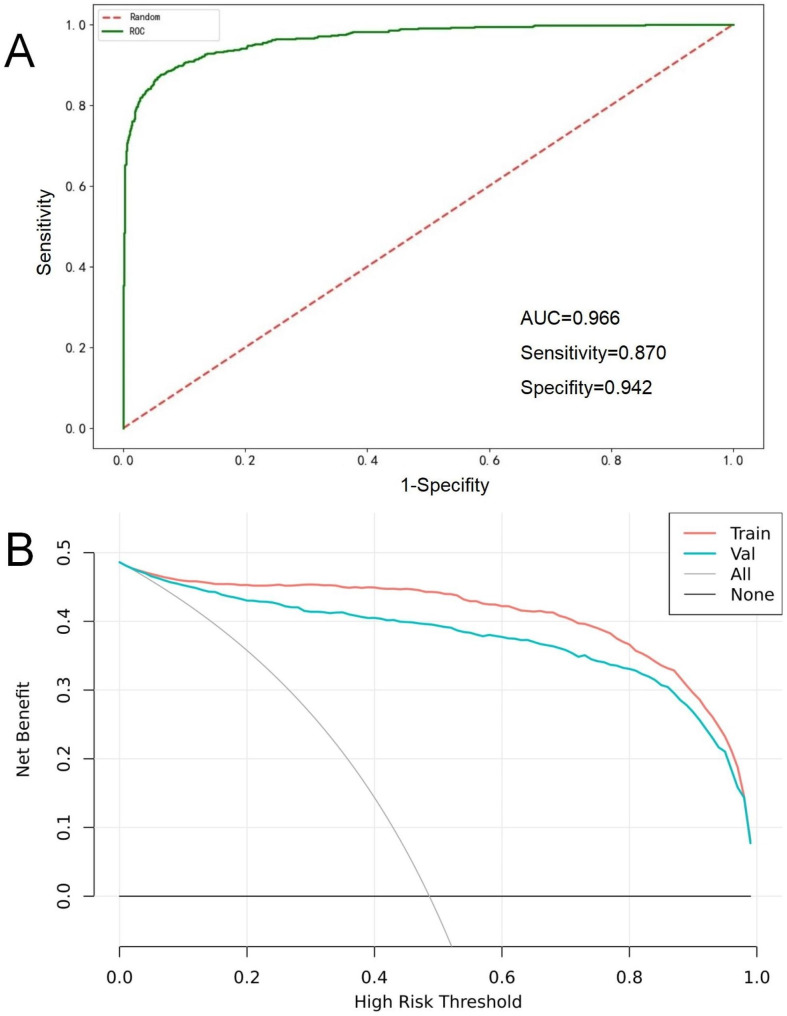
(**A**) The ROC curves of HF-Lab9 model constructed by machine learning XgBoost algorithm, (**B**) The DCA of HF-Lab9 model constructed by machine learning XgBoost algorithm

In cohort 2 First Hospital of Jilin University all enrolled study subjects from January 1, 2020, to December 31, 2021, the XgBoost algorithm was used to construct an HF warning model based on the above 9 model features, named HF-Lab9. The most important feature in the HF-Lab9 model was cTnI (Feature importance: 0.265), followed by TG, URBC, Urea, PAB, TT, γ-GT, GLU, and SG and in that order (Fig. 1). The distribution of the 9 model features among groups in cohort 2 is shown in Table 3. The ROC curve results showed that HF-Lab9 AUC = 0.966 in the validation set, and the decision curve analysis showed that both the training and validation sets HF-Lab9 showed high clinical benefits (Fig. 2).

External validation of the HF-Lab9 model was performed using 819 AMI patients from January to December 2022, including 398 patients who had a heart failure event.ROC curve results showed AUC = 0.94 [95%CI = 0.9237–0.9577], sensitivity = 0.849, and specificity = 0.914.

## Discussion

With the continuous development of society, people’s dietary habits have changed significantly and the stress of life has increased significantly, resulting in a gradual increase in the incidence of AMI. HF is one of the major adverse cardiovascular events after the onset of AMI. In recent years, most countries have experienced an aging population [13], HF is considered a major aging-related disease [14, 15], and the mean age of the study subjects in this study was greater than 60 years. However, except for heart transplantation for end-stage HF, there is no curative treatment available [16]. Accurately predicting the occurrence of HF after AMI remains a daunting task for clinicians because of the complex individualized variation exhibited among patients.

As electronic health records become more common, a large amount of information on patient visits is retained in hospital information systems around the world, providing suitable conditions for the application of ML. Traditional regression methods have difficulty in effectively handling high-dimensional interaction information in large datasets, which mechanistically limits the ability of models to predict complex relationships, and ML can overcome these difficulties. When dealing with complex data relationships, ML does not require assumptions about the type of data distribution and linear or non-linear relationships between features. ML can help identify potential predictor variables and improve the predictive accuracy of the model by modeling with computationally intensive iterative algorithms rather than manually selecting features [17]. In recent years, ML has been increasingly used in cardiovascular medicine, especially for patients with HF. For example, ML has been applied to the diagnosis of HF, mortality prediction, and readmission rates, with good performance [18, 19]. Previous studies have confirmed the excellent ability of random forest models in identifying risk factors in patients with HF and have successfully identified left ventricular ejection fraction as the most relevant feature in predicting the risk of death in patients [20]. Random Forest algorithm is a reliable method to improve the prediction accuracy of HF using a combined model containing 4 features [21]. In addition, the logistic regression model has been widely used in the prediction of HF in recent years and has performed well [22, 23]. XgBoost outperformed LR, RF, and support vector machines in predicting the incidence of heart failure and non-metastatic cervical cancer with AUCs of 0.8409 and 0.8365, respectively [24, 25]. In addition, XgBoost stood out among six ML algorithms in predicting lymph node metastasis in laryngeal cancer patients [26]. In this study, seven ML algorithms were evaluated, and the XgBoost algorithm stood out among the seven algorithms, demonstrating that it has the best predictive power for specific populations. The XgBoost algorithm has performed best in many studies probably because of its advantages as an integrated ML algorithm based on decision trees with fast computation, maximized prediction performance, minimized model complexity, and low overfitting [27].

Current HF diagnosis and management rely on physical examination, including laboratory and imaging data of patients [28]. In this study, we developed a model based on laboratory data for a new composite index, HF-Lab9, for predicting the risk of developing HF after AMI. cTnI was the first-ranked feature in HF-Lab9 in terms of importance, and cTnI levels were significantly higher in the HF group than in the control group. High-sensitivity cTnI is a predictor of mortality and vascular events in patients after ischemic stroke, and elevated high-sensitivity cTnI increases the risk of adverse cardiovascular and cerebrovascular events [29]. In addition, high-sensitivity cTnI has significant prognostic value in patients with non-ischemic HF, which can further significantly improve risk stratification and prediction in patients with non-ischemic HF [30]. In the model of this study, TG and GLU were also predictors of the development of HF after AMI. Studies show that high levels of triglycerides and cholesterol levels are risk markers for the late development of HF [31]. After myocardial infarction in non-diabetic patients, elevated blood glucose levels on admission are associated with the risk of developing HF [32]. Urea might be a possible biomarker of hormonal activation in the neurohumoral system of patients with HF [33]. Urea has been shown to be an important correlate of death after heart failure [34]. There is substantial evidence that elevated γ-GT activity is associated with an increased risk of cardiovascular diseases, such as HF and arrhythmias, but the evidence for an association with myocardial infarction is weaker. Therefore, γ-GT can be a valid predictive marker after the development of HF in patients with AMI [35]. Compared with past studies [36, 37], this study demonstrates that this novel composite index has a unique and high predictive ability for mortality risk in this specific population, providing a reliable assessment of the risk of developing HF after AMI.

Several advantages exist in this research study. First, the model included in this study has a wide range of features to be selected, with more than 70 routine tests, and effective references are provided in the modeling of screening and selection variables, such as modeling in pharmacology [38, 39] and genomics. In addition, the model is simple and easy to use, making secondary use of the huge amount of test data deposited by hospitals without adding additional economic burden. Second, the test data used in the model are all test results from the first admission of AMI patients, and the model has excellent advanced prediction capability, which provides sufficient lead time for preventing the occurrence of HF after AMI. In addition, the model was repeatedly validated with two cohorts containing four years of data, and the results were highly consistent and accurate. Third, the variety of ML algorithms evaluated in this study for comparison is large, which provides a good foundation for future model building. At the same time, there are some limitations to this study. First, this is a single-center cohort study, and the study population represents only one region and needs to be validated in multiple regions and multiple countries. Second, because the average age of the study population was older than 60 years, it belonged to the elderly population. The elderly population usually takes some medications, which may affect the model. Third, the characteristics to be selected in this study were limited to clinical tests and did not include electrocardiogram, imaging, and other findings.

This study compares seven ML algorithms to mine and examines the test big data, and finally the HF-Lab9 model containing 9 features of cTnI, TG, URBC, Urea, PAB, TT, γ-GT, GLU, and SG were constructed using the XgBoost algorithm. This model has high predictive efficacy and clinical benefit, provides a reliable model for predicting the risk of HF in AMI patients in clinical settings and evaluates multiple model-building algorithms for clinical prediction models.

## Data Availability

The datasets used and analysed during the current study available from the corresponding author on reasonable request.
